# District nursing staff’s experiences with implementing an evidence-informed triage instrument for delirium in home care; an action research-informed qualitative study

**DOI:** 10.1186/s12912-026-05028-6

**Published:** 2026-08-11

**Authors:** Karin E. van Os, Nina G. Hovenga, Henriette van der Kloet, Hendrika J. Luijendijk, Marike C. Schokker

**Affiliations:** 1 Department of Primary and Long-term Care, University of Groningen, University Medical Centre Groningen, Postbus 196, Groningen, 9700AD the Netherlands; 2 Kwadrant, Drachten, the Netherlands

**Keywords:** District nursing, Screening, Delirium, Research implementation, Aging, Action research, Clinical judgement

## Abstract

**Background:**

Timely recognition of delirium in people living at home remains challenging, despite the availability of evidence-informed approaches. District nurses and nursing assistants play a key role in translating such evidence into daily practice. This study aimed to explore how district nurses and nursing assistants experienced using and implementing the Delirium Caregiver Questionnaire (DCQ), an evidence-informed delirium triage tool, in routine home care.

**Methods:**

An action research-informed qualitative study was conducted with fifteen participants (district nurses and nursing assistants) from six district nursing teams. Implementation comprised three phases: preparation, training, and application. After three months of use, team-based interviews were conducted to explore experiences with the DCQ. Data were analysed inductively using thematic analysis.

**Results:**

Following 28 baseline assessments, the DCQ was used only four times during the three-month implementation period. Participants reported that the DCQ supported more structured delirium recognition by guiding observation, analysis, communication, and evaluation. Facilitators included the tool’s user-friendliness, collaborative engagement and commitment to client well-being. Barriers included stressful working conditions, not being ingrained in practice, a low sense of urgency, and limited opportunities to assess delirium.

**Conclusions:**

The DCQ was perceived as clinically valuable by supporting more structured delirium recognition and strengthening clinical reasoning. Despite strong perceived facilitators, integration into routine practice was limited by contextual and organisational constraints. Future research should evaluate targeted delirium screening in high-risk situations and explore how tools such as the DCQ can be embedded into routine workflows and electronic health records to support consistent use and timely consideration of delirium during clinical reasoning.

**Supplementary Information:**

The online version contains supplementary material available at https://doi.org/10.1186/s12912-026-05028-6.

## Background

Delirium is an acute neurocognitive condition characterized by cognitive dysfunction and a fluctuating course, typically triggered by underlying medical conditions, medication side effects, or physiological changes [1]. It is particularly prevalent among older adults with functional impairment or multimorbidity. Dementia is one of the strongest predisposing factors for delirium, and even relatively minor physiological disturbances may precipitate delirium in this population [2]. Delirium is linked to long-term cognitive decline, psychological distress, prolonged hospitalisation, functional impairment, institutionalisation, and higher mortality [2, 3].

Despite its impact, delirium frequently goes undetected in institutional and outpatient settings, with missed diagnoses estimated at 32%-76% [4–6]. In community settings, studies relying solely on clinical diagnoses similarly report the lowest prevalence rates, suggesting substantial underdetection when no structured diagnostic or screening instrument is used [7, 8]. The gap between available knowledge and clinical practice is particularly regrettable given that many underlying causes of delirium are treatable [9, 10].

Early recognition of delirium in community-dwelling older people is necessary to initiate timely treatment and minimise the impact of delirium. Home-managed delirium tends to be less severe and of shorter duration than delirium managed following hospitalisation [11]. Moreover, population ageing and policies encouraging older adults to live at home for as long as possible lead to higher rates of multimorbidity in this population [12], increasing the risk of delirium. In particular, frail older people receiving district nursing care or consulting a general practitioner (GP) and with cognitive impairment are vulnerable to delirium [8, 13]. Delirium is also particularly challenging to recognise in people with cognitive impairment, making timely recognition in this group especially important.

District nurses and nursing assistants (hereafter referred to as “nursing staff”) are well positioned to play a pivotal role in recognising delirium. Their frequent client contact enables them to report crucial observations regarding changes in a client’s condition to GPs [14, 15]. In this way, they act as a bridge between a client’s personal context, care needs, and other healthcare professionals [16].

Despite district nursing staff’s strategic position in caring for their clients, evidence indicates they often lack the knowledge and skills to recognise delirium effectively [17–19]. Additionally, applying evidence-based delirium recognition in outpatient settings faces contextual factors: nursing staff is not continuously present, symptoms can fluctuate and be subtle, and care is provided in the home environment – a high signal-to-noise environment embedded in daily life. As a result, nursing staff must distinguish clinical signals from a complex mix of factors, including both biomedical cues and dynamic, often ambiguous social environments [20, 21].

In institutional settings, evidence-based delirium recognition commonly relies on frequent screening using instruments such as the Confusion Assessment Method (CAM) or the Delirium Observation Screening Scale (DOSS) [22]. However, these tools have not been validated for community populations and can be labour-intensive [23]. From an evidence-based practice perspective, there is a need for a rapid screening approach to support standardised assessment and reinforce clinical awareness without placing undue burden on staff [17, 19, 24]. The Delirium Caregiver Questionnaire (DCQ) may address this gap.

The DCQ is a short, easy-to-use seven-item triage instrument that is completed by a healthcare professional and incorporates (in)formal caregiver observations [25] (see Additional file 1). It combines items reflecting both symptoms and known predictors of delirium to identify individuals who may require further evaluation for delirium. The DCQ can be applied without specific training and has been validated in older patients referred for dementia screening to an outpatient memory clinic at a mental healthcare institution [25]. As the instrument was validated in a population with cognitive impairment, it may be particularly relevant for use in cognitively impaired older adults receiving home care. Therefore, the DCQ may offer an evidence-informed approach to support delirium recognition by district nursing staff in this high-risk population.

However, the successful translation of evidence-informed tools into routine nursing practice depends on a range of factors, including organisational context and perceived acceptability, fidelity, feasibility, scalability and sustainability [26]. Implementation research consistently emphasises the value of tailored, context-specific approaches [27, 28], while real-world studies show wide variability in uptake and completion rates [29]. Moreover, implementation dynamics in home-care settings may differ from inpatient contexts, underscoring the need to understand how evidence-informed tools are experienced and integrated in district nursing practice [30].

## Methods

### Design and setting

This study aimed to explore how district nurses and nursing assistants experienced using and implementing an evidence-informed delirium triage tool (DCQ) in their daily practice.

The study used an action research-informed qualitative design, as implementing the DCQ in home care is a workflow-embedded, inherently context-dependent practice change. The design supported iterative cycles of planning, implementation and structured reflection to refine procedures in situ and prioritise feasibility and acceptability in routine care [31]. Nursing staff contributed to local tailoring and data collection, while the research team conducted analysis and interpretation. This practice-proximal, researcher-led approach enabled collaboration with nursing staff and context-specific adjustments across sites. The study is reported in accordance with the Consolidated Criteria for Reporting Qualitative Research (COREQ) guidelines [32] (see Additional file 2).

The study was conducted within Dutch district nursing, where district nurses and nursing assistants work together to provide community-based care. District nurses are primarily responsible for assessing care needs, developing care plans and coordinating care, whereas both professional groups provide direct client care and contribute to recognising changes in clients’ health status. Task allocation may vary between organisations and teams. The study was performed within the University Elderly Care Network of the University Medical Centre Groningen (UECN-UMCG). We aimed to recruit at least two district nursing teams and 30 home care clients.

### Recruitment

First, we approached contact persons (nurses and managers) from five home care organisations via meetings and email. We informed them about the purpose and method of the study and asked them to assess the availability and willingness of a district nursing team to participate. Ultimately, six teams from three organisations were recruited.

Next, within each team, champions were appointed who voluntarily took on a leading role in promoting and facilitating the implementation of the DCQ. Their responsibilities included initiating the first DCQ assessment, serving as the primary contact for team members and researchers, and motivating colleagues through reminders and discussing delirium during team meetings.

Finally, participating nursing staff identified clients who they considered to meet the study inclusion criteria and approached them in person during a home visit. Nursing staff informed potential participants about the study and obtained written informed consent from participants (or, where appropriate, from a legal representative). In total, 28 clients were approached, all consented and were enrolled.

### Inclusion criteria

To be eligible, a team had to provide district nursing care, defined in the Dutch context as professional nursing care delivered at home. This includes any technical, medical, supportive or rehabilitative nursing intervention, or assistance with personal care for (older) people living at home [33]. Care was delivered during routine home visits based on an individual care plan established following assessment by a district nurse. The care had to be provided under either the Dutch Health Insurance Act (Zorgverzekeringswet) or the Chronic Care Act (Wet langdurige zorg, WLZ).

Clients were eligible if the participating nursing staff perceived cognitive decline, regardless of formal diagnosis, and that the client received home care from one of the participating district nursing teams. No additional exclusion criteria were applied.

### Implementation procedures

To facilitate its use in Dutch district nursing, the presentation and supporting materials of the DCQ were adapted, while the questionnaire items themselves remained unchanged. Specifically, an instruction sheet was added with practical guidance on when and how to administer the DCQ. In addition, two action-oriented recommendations were added to guide follow-up after completion: ‘Continue care as usual’ for low-risk outcomes and ‘Contact the general practitioner’ for high-risk outcomes. These recommendations were intended to support, rather than replace, nursing assessment and clinical decision-making following completion of the DCQ.

The implementation of the DCQ consisted of three phases: preparation (two months), training (one month), and application (three months). During the preparation phase, one or two researchers (KO and/or MS) and a nursing-trained implementation expert (HvdK) met with the champions to tailor the workflow, materials, implementation approach and action plan to each team’s way of working. Discussion topics and the structure of the action plan (Additional file 3) were drawn from established implementation models [34, 35]. Tailoring to local context included decisions regarding who would apply the DCQ (champions only versus all nursing staff) and the set-up of the champion group.

In the training phase, all participating nurses and nursing assistants were encouraged to complete an organisational online learning module on delirium. Additionally, experienced educators with backgrounds in education or nursing provided a two-hour educational training session (Additional file 4). As the DCQ can be applied without specific training, the educational session focused primarily on delirium knowledge and recognition (signs, prevalence, consequences, and recognition strategies), and provided only a brief practical introduction to the DCQ. The target of at least 60% staff participation in the educational session was achieved in all participating teams. Following the training, participants conducted a baseline DCQ assessment in 28 clients to familiarise themselves with its application.

Finally, during the application phase, the nursing staff applied the DCQ in routine home visits based on their clinical judgement or when they experienced a ‘gut feeling’. Clinical judgement referred to their assessment whether further evaluation was warranted. Gut feeling referred to a sense that something was wrong or that the client was ‘different from usual’, despite being unable to immediately explain the underlying reason for this concern. In some teams, champions applied the DCQ following consultation, whereas in others all nursing staff used the tool.

### Data collection

We conducted semi-structured group interviews with the teams to investigate their experiences with the DCQ. The interviews took place approximately three months after training and completion of the application phase.

A semi-structured interview guide was developed based on both the Consolidated Framework for Implementation Research (CFIR) [36], which focuses on implementation factors, and the Reach, Effectiveness, Adoption, Implementation, and Maintenance (RE-AIM) model [37] to assess the outcomes. Relevant CFIR constructs and topic areas were informed by key strategies, facilitators and barriers to implementation in community nursing identified in a systematic review of mixed-methods studies [38].

The group interviews began with an open-ended question that allowed participants to describe their experiences with the DCQ (Additional file 5). Subsequent questions addressed: (1) preparations for DCQ use, (2) experiences and outcomes, (3) use in practice, (4) suitability for older people living at home, (5) miscellaneous influencing factors, and (6) future application and recommendations.

Two female researchers (KO and MS) with no prior relationship with the participants conducted six post-implementation group interviews – one per team – with 15 participants (nursing staff). KO, a registered nurse and PhD candidate with a background in district nursing, had received training in qualitative interviewing. MS held a PhD and was an experienced qualitative researcher. Prior to the interviews, participants were informed about the purpose of the study and the professional backgrounds of the interviewers. Five interviews took place at the workplace and one online at participants’ request. Interviews lasted 30–80 min. A non-participant (a toddler) was present during the online interview. Field notes were made during and immediately after the interviews, directly followed by researcher debriefing.

In addition, data on actual DCQ use were collected by extracting completed DCQs, including assessment timing (baseline or follow-up), total scores, and nursing staff’s rationale for follow-up assessment.

### Data analysis

We audio recorded and transcribed the group interviews verbatim, followed by pseudo-anonymisation of the transcripts (removing names and personal characteristics while retaining roles and occupations). We utilised ATLAS.ti version 24.1.1.30813 [39] for data management. Data were analysed using Braun and Clarke’s thematic analysis [40] with an inductive coding approach.

Two authors (KO and MS) independently performed open coding on two interviews, and subsequently discussed the codes to reach consensus and develop a codebook. KO applied the codebook to the remaining interviews, adding new codes where necessary. All coded interviews and newly added codes were reviewed by MS and discussed until consensus was reached. This iterative process ensured that themes and subthemes accurately reflected the data. Finally, three researchers (KO, MS and NH) rigorously discussed and refined the themes and subthemes, and extracted authentic quotes to support the findings [41].

### Ethical considerations

The Medical Ethics Review Committee of the University Medical Centre Groningen (UMCG) granted ethical approval for the study (202100589) on the 12th of May 2022 and confirmed that the Medical Research Involving Human Subjects Act (WMO) did not apply to our study. All procedures performed in this study involving human participants were conducted in accordance with the 1964 Declaration of Helsinki and its later amendments or comparable ethical standards. Participating clients were included after giving written informed consent.

### Rigor and reflexivity

Scientific rigour was achieved by addressing questions of credibility, dependability, and transferability throughout the study [42]. Credibility was strengthened through peer debriefing focused on codes, themes, and subthemes striving for consensus. We conducted a member check by sending a short summary of the findings to participants and asking whether they recognised and agreed with the interpretation of their input. Triangulation was achieved by collecting data from nursing staff across different teams and comparing interview data with records of actual DCQ use in practice.

Dependability was supported by maintaining an audit trail documenting methodological decisions and analytical decisions. Transferability was addressed by providing a detailed description of the study setting, participant characteristics, and context, enabling readers to assess applicability to other community-based nursing settings.

## Results

Six district nursing teams (teams A-F) participated, from three healthcare providers in the UECN-UMCG. Team size varied from 5 to 20 nursing staff employees, serving 20–50 clients per team. Each team designated one to four champions. One team discontinued the implementation due to champions’ workload but still participated in an interview.

Fifteen healthcare professionals were interviewed, including fourteen women and one man: six district nurses, eight nursing assistants and one team manager (see Additional file 6). Based on the interviews, we identified three main themes and ten subthemes and are presented below (Table 1).

In total, 28 clients who met the study criteria participated (0–10 per team). All had (perceived) cognitive decline. A baseline DCQ assessment was completed for all 28 clients upon study enrolment, and all baseline assessments were negative for delirium. During the three-month application period, the DCQ was applied four additional times: three assessments involved participating clients, and one was performed by a participating nurse for a client from another team. All four assessments were positive and prompted consultation with the GP, in accordance with the DCQ work instructions. Delirium was considered probable in all screen-positive clients by the consulted GP. However, based on follow-up communication with the GP, family members or colleagues, participating nursing staff was not aware of any comprehensive medical assessment having been conducted.

**Table 1:** Main themes and subthemes

Main theme	Subtheme
Clinical value	- Structured thinking and action - Improved communication - More opportunities for early treatment
Facilitators to adoption	- User-friendly - Collaborative engagement - Commitment to client well-being
Barriers to adoption	- Stressful working conditions - Not ingrained in practice - Low sense of urgency to use the DCQ - Limited opportunities to assess delirium

### Clinical value

Participating nursing staff was generally positive about the (potential) use of the DCQ and expressed interest in continued use beyond the project. They reported that the DCQ supported more structured recognition by aiding observations, guiding analyses, supporting communication and prompting evaluation, thereby facilitating earlier treatment.

#### Structured thinking and action

Most participants described that the DCQ introduced a more structured approach to delirium detection. It helped substantiate initial impressions or ‘gut feelings’, prompting critical thinking and a broader appraisal of the patient’s situation. Rather than relying solely on a general sense that “something was wrong”, the questionnaire encouraged nurses to substantiate their concerns. It offered specific observation points and guidance for targeted questioning. As one nurse noted: *“I think colleagues often have a gut feeling that something isn’t right, but they cannot always substantiate it. With this questionnaire you start asking much more specifically what has changed. Has someone become more confused? Are they restless during the night? These are much more factual questions, allowing you to support your observations”* (R2 – nurse).

Participants explained that completing the DCQ extended beyond simply answering the questions. Working through the questionnaire helped them rule out alternative explanations, reflect on their observations, and determine appropriate follow-up actions. One nursing assistant illustrated: “*It is important not to take things at face value but gather more facts and carefully think about what could be going on. It is such a clear questionnaire – you think: okay, I ruled all of that out. Then I reflect with the district nurse to determine the next step?* […] *That’s the strength of it. You don’t just look at the delirium test, but you look further*” (R1 – nursing assistant).

Several participants described that the DCQ encouraged team discussions and evaluation, functioning as a shared informal checklist. One nurse explained: *“It has helped because delirium is much more on our radar now. We discuss it with each other: ‘We’re seeing this—could this be delirium? What are the symptoms again?’ It really helped to keep delirium in everyone’s attention”* (R14, nurse).

Overall, nursing staff emphasised that the value of the DCQ lay in the way it stimulated structured clinical reasoning rather than in completing the questionnaire itself.

#### Improved communication

Many participating nursing staff believed that the DCQ could improve communication with colleagues and GPs, which they viewed as crucial for delirium recognition. They reported that the tool helped structure and clarify case presentations, increasing GP responsiveness. One nursing assistant explained: “*When you call a GP, you keep it as concise as possible and focus only on the facts. You are taken much more seriously* [.], *it appears more professional, and the GP will take more action*” (R7 – nursing assistant). The DCQ also legitimised initiating contact with the GP for some nursing staff.

Participants also reflected on situations in which communicating concerns about delirium to GPs had been challenging. One nurse described the frustration of repeatedly raising the possibility of delirium while feeling that these concerns were not acknowledged: *“It was just frustrating that we had to fight so hard to convince the GP. We kept saying: he has a delirium. Could you please focus on that? […] That lasted for almost 14 days”* (R8 – nurse).

Communication with clients was discussed less frequently but was also considered relevant. Some participants explained that using the DCQ created an opportunity to explore changes in cognition and behaviour in greater depth. As one nursing assistant noted: “*The questionnaire gave you an opening. Normally you’re busy providing care and you don’t really have that conversation because you don’t have the time.* [.] *With the questionnaire, and by explaining why we were using it, you learned much more about people” (*R7 – nursing assistant). At the same time, several participants indicated that, in routine practice, they would not administer the questionnaire as a formal interview but would integrate the questions into everyday care encounters. As one participant explained:

*“If I suspected delirium, I would ask these questions while helping someone shower or get dressed. It would happen much more naturally”* (R9 – nursing assistant).

#### More opportunities for early treatment

Almost all participating nursing staff emphasised the importance of timely delirium recognition, as it enables timely intervention, prevents deterioration and supports faster recovery. Several participants expressed regret about missed opportunities for timely action, especially since underlying causes of delirium are sometimes easily treatable. One nurse explained: *Recognising delirium early, means that treatment can start sooner, and the situation does not escalate* (R2- nurse).

### Facilitators to adoption

Participating nursing staff highlighted several facilitating factors that influenced the implementation of the DCQ positively.

#### User-friendly

All participating nursing staff described the DCQ as simple, quick and easy to use. Its flexible integration into daily workflows, enabled teams to adapt its use to their specific needs. The DCQ-items were generally considered clear. One nurse described: “*I think if you’ve had one training session and have the pocket card, you’ll manage. It’s not like you need to see a delirium client every week or you’ll forget how to use it*” (R10 – nurse). Several participants contrasted the DCQ with more formal cognitive screening tools, explaining that these could feel uncomfortable for clients, whereas the DCQ could be integrated more naturally into routine nursing care.

#### Collaborative engagement

All participating nursing staff valued the opportunity to contribute to the project and tailor the DCQ’s implementation to their team’s workflow. One nurse explained: “*Our questions and suggestions were actually listened to, which allowed us to truly work as a team (with the researchers)*” (R8 - nurse). Several participants emphasised the importance of a bottom-up involvement, as one nurse stated: “*Projects should not be imposed from above if you want to get people on board*” (R4 - nurse). The collaboration stimulated the teams to take a more active role in the implementation process. One nursing assistant shared: “*By being allowed to think along, we also started thinking about it in several ways. How are we going to do this?* (R6 – nursing assistant)”. However, participants emphasised that implementation could not depend on a single enthusiastic individual but required engagement across the team. As one team manager explained: *“You can’t do something like this on your own. You really have to do it as a team, and that motivation just wasn’t there”* (R5 – team manager). Reflecting on this experience, she added: *“If you involve people beforehand and take more time to get them enthusiastic, I think you have a better chance”* (R5 – team manager).

#### Commitment to client well-being

The participating nursing staff consistently expressed a strong sense of responsibility for ensuring the best possible care for their clients. As one nurse stated: “*We all aim for the best quality of care*” (R3 - nurse). Recognising delirium was seen as an essential part of preventing unnecessary suffering, although the DCQ was not always explicitly mentioned in this context.

Delayed or missed recognition of delirium was considered particularly problematic, as it could lead to incorrect treatment, prolonged symptoms and potentially hospital or nursing home admission. One nursing assistant noted: "*Relocation can be a drama for a client. Not every client copes well with change"* (R7 – nursing assistant).

### Barriers to adoption

Despite a generally positive perception of the DCQ, its utilisation remained limited, with only three instances of use across all teams beyond the initial baseline measurement. We identified several barriers to adoption.

#### Stressful working conditions

Participating nursing staff described a work environment characterised by understaffing and overburdened colleagues, which not only hindered delirium recognition but also limited the capacity to implement new tools or workflows. Team members already carried additional responsibilities, impacting their free time – making them reluctant to take on further tasks. One nursing assistant described: “*You work double shifts. You’re already so busy with work that when someone asks if you can focus on something extra, you think: please, don’t ask me. [.] Today is my day off. Yet, I’m here, and I must plan routes and rearrange everything because a colleague isn’t feeling well. This all comes out of my personal time. Some clients even joke, ‘Do you have a bed here?’ That’s how much we work. It really stands out*” (R7 – nursing assistant). Workload pressures were also evident during data collection, as four of six interviews were interrupted by work-related phone calls, underlining the ongoing pressure on staff. The lingering impact of COVID-19 was noted as a source of exhaustion of the team (R6 – nursing assistant).

Within this context, taking on the champion role was experienced as challenge, although all nursing staff recognised its importance. Team changes and overwhelming workload were seen as particularly detrimental, and in two teams remaining champions felt unable to carry the project alone. As one nursing assistant shares: “*As a champion, it is easier to do it with someone else […] My motivation decreased when no one wanted to help […] What can I do now to continue with this?”* (R6 – nursing assistant). Even when multiple champions were appointed, maintaining champion-related tasks proved difficult.

At the same time, some participants reflected critically on workload as a perceived rather than absolute barrier. One nurse reflects on this tension: “*You know, there’s so much. And I tell you, you quickly hide behind ‘it’s too busy’. But it always amazes me what is actually possible.*” (R3 - nurse). Another nursing assistant adds: “*You feel like you don’t have the time”* (R13).

#### Not ingrained in practice

Participants reported that using the DCQ had not yet become an automatic part of routine assessment practices and was therefore often overlooked. They reflected the DCQ should become a routine response when clients show signs of change, similar to standard assessments such as blood pressure or glucose levels. Looking back at clients’ care reports, several participants recognised situations in which the DCQ could have applied but had not been considered at the time. As one nurse reflected: *“When you look back afterwards, you think: we could have used it”* (R2 – nurse). Another participant elaborated: *“The gut feeling was there—that something was going on with this client. We just didn’t make the connection to this [the DCQ] […] If you have a gut feeling, a bell should ring to use the DCQ, similar to standard measurements when someone is unwell”* (R1 – nursing assistant).

#### Low sense of urgency to utilise the DCQ

Nursing staff perceived delirium as relatively uncommon among their clients and noted that behavioural changes were often recognised without using the DCQ. One nurse explained: *“Eight or nine out of ten times, from what I have experienced, I can recognise it without the DCQ. It is mainly useful in doubtful cases”* (R4 – nurse). Some participants remarked that the content of the DCQ items was already familiar, limiting the perceived added value of completing the tool in daily practice. Paradoxically, all nursing staff expressed concerns about potentially missing signs of delirium. One nurse even questioned whether delirium was truly as rare as perceived and reflected that behavioural changes were often attributed to dementia rather than considered as possible signs of delirium: *“We tend to associate confusion with cognitive problems. If someone has dementia, we think: ‘oh well, just a bit forgetful’. It is not immediately a trigger”* (R2 – nurse).

#### Limited opportunities to assess delirium

Practical constraints limited nursing staff’s ability to observe signs of delirium and complete the DCQ. Their presence was short and infrequent, described as “waai/fleeting moments” by one nurse: “*You come in and out as home care. You’re there only shortly. If you’re lucky and it’s a personal care visit, you’re there for 45 minutes or an hour”* (R3 - nurse). Understaffing also led to frequent client rotation among team members, reducing continuity of care and making subtle behavioural changes harder to detect. One nurse explained: *“If you don’t know a client, you are less likely to recognise abnormal behaviour”* (R14 - nurse).

Relevant information was often difficult to obtain, as most clients live alone and informal carers are only sporadically present. Clients themselves were seen as unreliable informants regarding their cognitive state, as one nurse reflected: *“How reliable is the answer you get from the client about changes in their day–night rhythm?”* (R2 – nurse). Family members were also frequently unavailable to provide information, as one nurse stated: *“Informal caregivers are simply no longer present or involved”* (R3 – nurse). These limitations were particularly pronounced at night – when delirium symptoms are often most visible, while most district nursing teams do not provide routine overnight care. R13 (nursing assistant), R14 (nurse) and their colleagues expressed that these information constraints made it difficult to apply the DCQ in home care, decreasing motivation to use the tool within their team.

## Discussion

In this action research-informed qualitative study, we explored how district nurses and nursing assistants experienced the use and implementation of an evidence-informed delirium triage tool (the DCQ) in their daily practice. These findings also help explain the limited uptake of the DCQ. Participating nursing staff generally valued the DCQ for supporting more structured delirium recognition, through observation, analysis, communication and evaluation. These processes align closely with core components of clinical reasoning in nursing literature [43].

Our findings underscore that tools such as the DCQ can support – but not replace – nurses’ clinical reasoning. Strong clinical reasoning is widely recognised as an essential nursing skill, vital to delirium recognition and high-quality nursing care, and is associated with improved patient outcomes [44, 45]. District nurses rely on clinical reasoning to interpret clinical observations, and the effective use of clinical tools likewise depends on nurses’ professional clinical competence [46, 47]. However, their value depends on recognising when behavioural changes should prompt consideration of delirium as a diagnostic possibility.

The added value of the DCQ may lie in directing attention to diagnostic cues for delirium while naturally fitting into routine care through its observational and conversational format. In everyday nursing practice, clinical reasoning often prioritises management-oriented considerations: patient preferences, long-term care goals, and contextual factors [20, 48]. This management emphasis may be particularly pronounced in home care, where nursing staff must navigate complex social and environmental dynamics. However, clinical reasoning for delirium recognition requires a strong diagnostic emphasis.

Successful implementation appeared to depend not only on the characteristics of the DCQ itself, but also on the way it was implemented within teams. Nursing staff described the DCQ as flexible to integrate and simple, quick and easy to use – characteristics known to support implementation of new practices in district nursing [38, 47]. Teams also valued the opportunity to co-design the implementation process, consistent with prior research [38]. Such collaborative engagement may be particularly effective in district nursing, where flexibility and professional autonomy are defining features: they not only provide care, but also organise and manage their own caseloads [49]. However, co-design alone appeared insufficient; successful implementation also required team-wide engagement.

Finally, the improvement in communication may facilitate implementation by making the added value of the DCQ immediately visible in daily practice. For example by helping communicate concerns about possible delirium more effectively, which may offer a tangible return on investment for adopting new practices in district nursing [38].

Despite the positive reception of the DCQ and the presence of facilitating factors, it had not been ingrained in practice, limiting actual use. Nursing staff applied the DCQ based on their own clinical reasoning rather than a standardised trigger, aligning with their autonomous ways of working. However, behavioural changes or gut feelings did not consistently prompt consideration of delirium. This may have contributed to the limited uptake, as tasks without a clear or consistent place in daily routines are more likely to be deprioritised or forgotten [50].

Nursing staff also described how the context of district nursing limited opportunities to observe behavioural changes and to implement the DCQ. Factors such as limited continuity of care, restricted opportunities to observe clients, and limited access to information reduced opportunities to recognise behavioural changes and to know the client – an aspect central to sound clinical reasoning and emphasised in previous literature [51]. These contextual barriers were compounded by structural challenges such as time constraints, high workload, and staff shortages, which hindered both delirium recognition and implementation of the DCQ. Similar barriers have been reported in other studies district nursing studies [38, 47], and reflect a persistent trend in district nursing [52, 53].

The champion role appeared to be constrained in the context of existing workload. Although champions are recognised as critical to driving change [54], many staff members were already burdened with additional tasks. These often extended into their personal time, leaving little willingness to assume further responsibilities. Consequently, champions in several teams reported feeling isolated or overwhelmed in their efforts to sustain momentum. These findings highlight the need for protected time and shared ownership to support champions in future implementation efforts.

Notably, nursing staff simultaneously expressed confidence that no delirium cases had been missed and an awareness that some were likely missed. This apparent paradox may reflect the unique context of district nursing. Limited feedback loops (e.g., confirmation from a GP or hospital) mean that missed cases often remain invisible, potentially reinforcing perceptions of adequate detection. At the same time, nursing staff’s clinical experience and awareness of diagnostic complexity may prompt concerns about missed delirium. Feedback is essential for improving clinical reasoning [55], as information on patient outcomes enables healthcare professionals to reflect on and improve their care processes [56].

### Strengths and limitations

This study has several strengths. First, it was conducted in a real-world community care setting, enhancing ecological validity – the extent to which findings align with real-world phenomena [57]. Embedding the study in district nursing teams’ existing workflows ensured that findings reflect the actual conditions and challenges of implementing evidence-informed care in the community, thereby increasing relevance for evidence-based practice. Second, the intervention was collaboratively tailored to each team’s way of working, enhancing contextual relevance and buy-in [58].

However, some limitations should be noted. First, all approached clients agreed to participate, suggesting potential selection at the approach stage (gatekeeping). Nursing staff may have prioritised clients with whom they had a good relationship or those perceived as more cooperative, rather than selecting clients purely based on (suspected) cognitive decline. Nevertheless, interviews solicited all experiences with the DCQ, and teams also recorded DCQ use among non-enrolled clients. This makes it unlikely that any consent-related selection substantially altered the findings. Second, while data saturation was not formally evaluated, interviews across six different teams involving 15 nurses and nursing assistants provided diverse perspectives. Thematic consistency across teams suggested that key implementation issues were adequately identified. Third, a longer implementation period would have allowed lessons learned during the initial implementation phase to inform subsequent implementation efforts. This may have provided additional insights into sustained adoption.

### Recommendations

We have several recommendations for future policy, practice and research. First, we recommend a bottom-up, collaborative approach – paired with targeted awareness-raising – to strengthen delirium recognition in home care. Co-designing implementation with teams aligns with the flexibility and professional autonomy of district nursing and may strengthen nursing staff’s intrinsic motivation. Targeted awareness-raising, for example through external feedback on delirium care, may further increase awareness, create urgency, and support implementation [59].

Second, strengthen nursing staff’s clinical reasoning skills and provide explicit triggers for delirium assessment. Strengthening diagnostic reasoning alongside the contextual and management-oriented reasoning required in community care may improve evidence-based nursing practice. Tools such as the DCQ support may help embed attention to diagnostic cues within routine clinical reasoning.

Third, future research should evaluate targeted delirium screening approaches in high-risk situations and explore how tools such as the DCQ can be embedded into routine workflows and electronic health records to support consistent use. High-risk situations may include post-hospitalisation, sudden behavioural changes, new medication regimens, or suspected infections. Targeted screening may balance feasibility and early detection. For instance, automated data-driven prompts in high-risk scenarios could trigger screening. Improving recognition rates could boost perceived relevance, counter the perception that delirium is rare and create opportunities for timely preventive interventions.

Lastly, although not a new issue, it is crucial to address the impact of staff shortages. Barriers identified in this study – such as lack of continuity, short visits, high workload, and insufficient staffing – require urgent and structural attention. These barriers not only affect day-to-day care delivery but also obstruct the uptake of evidence-informed practices. Effective implementation therefore requires structural support, including adequate staffing, protected time for champions and clearly defined team roles to prevent burnout and ensure continuity.

## Conclusions

This study highlights the clinical value of the Delirium Care Questionnaire (DCQ) as a structured approach to delirium recognition in district nursing. Nursing staff valued the tool for supporting clinical reasoning through structured observations, communication, and evaluation. Adoption was supported by the tool’s user-friendly design, collaborative engagement and a commitment to high quality, client-centred care. However, integration into routine practice remained limited due to contextual barriers, including high workload, fragmented care, and limited continuity. Future research should evaluate targeted delirium screening in high-risk situations and explore how tools such as the DCQ can be embedded into routine workflows and electronic health records to support consistent use and timely consideration of delirium during clinical reasoning.

## Supplementary Information

Below is the link to the electronic supplementary material.

Supplementary Material 1: Additional file 1. DCQ; Additional file 2. Consolidated criteria for reporting qualitative studies (COREQ): 32-item Checklist; Additional file 3. Action plan; Additional file 4. Educational session; Additional file 5. Interview guide; Additional file 6. Team-specific details

## Data Availability

The datasets generated and/or analysed during the current study are not publicly available due to privacy issues but are available from the corresponding author on reasonable request.

## Abbreviations

DCQ: Delirium Caregiver Questionnaire
GP: General practitioner
