# Supplementary material for: District nursing staff’s experiences with implementing an evidence-informed triage instrument for delirium in home care; an action research-informed qualitative study
Source: BMC Nurs. 2026 Aug 11;25:1084. doi: 10.1186/s12912-026-05028-6 (PMC13741438; doi:10.1186/s12912-026-05028-6)
Supplement: Supplementary file 1 [file 12912_2026_5028_MOESM1_ESM.docx]

**Online Additional file**

**District nurses’ experiences with implementing an evidence informed triage instrument for delirium in home care; an action research-informed qualitative study**

**Content**

1. Additional file 1. DCQ, page 2
2. Additional file 2. Consolidated criteria for reporting qualitative studies (COREQ): 32-item Checklist, page 4
3. Additional file 3. Action plan, page 8
4. Additional file 4. Educational session, page 11
5. Additional file 5. Interview guide, page 13
6. Additional file 6. Team-specific details, page 15

**Additional file 1. DCQ**

*Manuscript:* District nurses’ experiences with the implementation of an evidence-informed triage instrument for delirium in home care; a qualitative study

**Additional file 2. Consolidated criteria for reporting qualitative studies (COREQ): 32-item Checklist** *Manuscript:* District nurses’ experiences with the implementation of an evidence-informed triage instrument for delirium in home care; a qualitative study

Developed from:
Tong A, Sainsbury P, Craig J. Consolidated criteria for reporting qualitative research (COREQ): a 32-item checklist for interviews and focus groups. *International Journal for Quality in Health Care*. 2007. Volume 19, Number 6: pp. 349 – 357

| No. Item | Guide questions/Description | Reported |
| --- | --- | --- |
| **Domain 1: Research team and reﬂexivity** | | |
| *Personal Characteristics* | | |
| 1.Interviewer/facilitator | Which author/s conducted the interview or focus group? | 2.5 Data collection Page 8 |
| 2. Credentials | What were the researcher’s credentials? e.g. PhD, MD | 2.5 Data collection Page 8 |
| 3. Occupation | What was their occupation at the time of the study? | 2.5 Data collection Page 8 |
| 4. Gender | Was the researcher male or female | 2.5 Data collection Page 8 |
| 5. Experience and training | What experience or training did the researcher have? | 2.5 Data collection Page 8 |
| *Relationship with participants* | | |
| 6. Relationship established | Was a relationship established prior to study commencement? | 2.5 Data collection Page 8 |
| 7.Participant knowledge of the interviewer | What did the participants know about the researcher? e.g. personal goals, reasons for doing the research | 2.5 Data collection Page 8 |
| 8. Interviewer characteristics | What characteristics were reported about the interviewer/facilitator? e.g. Bias, assumptions, reasons and interests in the research topic | 2.5 Data collection Page 8 |
| **Domain 2: study design** | | |
| *Theoretical framework* | | |
| 9. Methodological orientation and Theory | What methodological orientation was stated to underpin the study? e.g. grounded theory, discourse analysis, ethnography, phenomenology, content analysis | 2.5 Data collection Page 8 2.6 Data analysis Page 9 |
| *Participant selection* | | |
| 10. Sampling | How were participants selected? e.g. purposive, convenience, consecutive, snowball | 2.2 Recruitment Page 6 |
| 11. Method of approach | How were participants approached? e.g. face-to-face, telephone, mail, email | 2.2 Recruitment Page 6 |
| 12. Sample size | How many participants were in the study? | 3 Results Page 10 |
| 13. Non-participation | How many people refused to participate or dropped out? Reasons? | 3 Results  Page 10 |
| *Setting* | | |
| 14. Setting of data collection | Where was the data collected? e.g. home, clinic, workplace | 2.5 Data collection Page 8 |
| 15. Presence of non-participants | Was anyone else present besides the participants and researchers? | 2.5 Data collection Page 8 |
| 16. Description of sample | What are the important characteristics of the sample? e.g. demographic data, date | 3 Results  Page 10 Additional file 4 |
| *Data collection* | | |
| 17. Interview guide | Were questions, prompts, guides provided by the authors? Was it pilot tested? | 2.5 Data collection Page 8 Additional file 5 |
| 18. Repeat interviews | Were repeat interviews carried out? If yes, how many? | No. |
| 19. Audio/visual recording | Did the research use audio or visual recording to collect the data? | 2.6 Data analysis Page 9 |
| 20. Field notes | Were ﬁeld notes made during and/or after the interview or focus group? | 2.6 Data analysis Page 9 |
| 21. Duration | What was the duration of the interviews or focus group? | 2.5 Data collection Page 8 |
| 22. Data saturation | Was data saturation discussed? | 4.1 Strengths and limitations Page 21 |
| 23. Transcripts returned | Were transcripts returned to participants for comment and/or correction? | 2.8 Rigor and reflexivity Page 9 |
| **Domain 3: analysis and ﬁndings** | | |
| *Data analysis* | | |
| 24. Number of data coders | How many data coders coded the data? | 2.6 Data analysis Page 9 |
| 25. Description of the coding tree | Did authors provide a description of the coding tree? | 3 Results Page 11 |
| 26. Derivation of themes | Were themes identiﬁed in advance or derived from the data? | 2.6 Data analysis Page 9 |
| 27. Software | What software, if applicable, was used to manage the data? | 2.6 Data analysis Page 9 |
| 28. Participant checking | Did participants provide feedback on the ﬁndings? | 2.8 Rigor and reflexivity Page 9 |
| *Reporting* | | |
| 29. Quotations presented | Were participant quotations presented to illustrate the themes/ﬁndings? Was each quotation identiﬁed? e.g. participant number | 3 Results page 12-18 |
| 30. Data and ﬁndings consistent | Was there consistency between the data presented and the ﬁndings? | 3 Results page 10-18 |
| 31. Clarity of major themes | Were major themes clearly presented in the ﬁndings? | 3 Results page 10-18 |
| 32. Clarity of minor themes | Is there a description of diverse cases or discussion of minor themes? | 3 Results page 10-18 |

**Additional file 3. Action plan**

*Manuscript:* District nurses’ experiences with the implementation of an evidence-informed triage instrument for delirium in home care; a qualitative study

Developed using principles from Grol & Wensing and Kotter’s change management frameworks, by the implementation experts of the University Elderly Care Network of the University Medical Centre Groningen (UECN-UMCG).

This action plan was used during the preparation phase of the intervention to guide implementation of the Delirium Care Questionnaire (DCQ) in district nursing teams. It is based on evidence-informed elements drawn from implementation science and change management theory, particularly the frameworks developed by Grol & Wensing (2006) and Kotter (1995). The plan was tailored collaboratively with each participating team to ensure contextual fit.

## **1. General Conditions and Initial Preparation**

| Item | Action | Operationalisation |
| --- | --- | --- |
| Assess time and resource availability | Determine whether team members have time and capacity to participate in the implementation. | All teams: team manager of team coach discusses this with team. Only continue with confirmation that there is enough time and capacity. |
| Leadership support | Secure formal endorsement from management (e.g., board or team coach), and communicate this support clearly to the team. | All teams: have meetings with director and local team manager or team coach.  Team manager or coach communicates their endorsement verbally and through written communication. |
| Define project roles | Clarify who is part of the implementation team and what their responsibilities are. | All teams: agree who are the local champions and agree upon what this entails. |

## **2. Stakeholder Mapping and Involvement**

| Item | Action | Operationalisation |
| --- | --- | --- |
| Identify target users | Define which professionals will use the DCQ (e.g., nurses, nurse assistants). | In all teams: nurses and nurse assistants |
| Identify affected stakeholders | Include clients, dementia case managers, and informal caregivers where appropriate. | Nursing staff will contact clients with (perceived) cognitive decline to inform them about the study and ask them to participate.  Researchers provide information letters and informed consent forms.  Nursing staff will give filled in forms to researchers. |
| Inform key partners | Consider sending information letters to GPs and case managers to align expectations and communication. | Champions will send information letters to GPs and case managers.  Researchers provide information letters. |

## **3. Building a Guiding Coalition**

| Item | Action | Operationalisation |
| --- | --- | --- |
| Form a leading team | Identify motivated team members who will act as local champions. Ensure they are credible, communicative, and committed. | Different starting compositions in the champion groups:  Team 1, 5: two nursing assistants and one district nurse  Team 2, 3: three nursing assistants and one district nurse  Team 4: two district nurses  Team 6: four nursing assistants |

## **4. Creating a Sense of Urgency**

| Item | Action | Operationalisation |
| --- | --- | --- |
| Share the why | Present data on the prevalence and impact of delirium, and the potential for early intervention through structured screening. | Present this during educational session |
| Clarify roles | Ensure clarity on what is expected from each role in the team during implementation. | Discuss this during educational session for entire team  Discuss this with champions during meetings |

## **5. Goal Setting**

| Item | Action | Operationalisation |
| --- | --- | --- |
| Define SMART goals | Set clear and measurable objectives for DCQ use (e.g., number of screenings, training completion). | Discuss objectives with champions during meetings |

## **6. Contextual Analysis**

| Item | Action | Operationalisation |
| --- | --- | --- |
| Assess barriers and facilitators | Identify factors that might hinder or support implementation (e.g., staff motivation, client willingness, time constraints). | Discuss this with champions during meetings |
| Tailor plan accordingly | Adjust intervention components to local routines and resources. | Discuss this with champions during meetings |

## **7. Training and Education**

| Item | Action | Operationalisation |
| --- | --- | --- |
| E-learning | Assign relevant e-learnings on delirium to participants. | Each team member completes their organizational e-learning |
| Live session | Deliver tailored, in-person training session on delirium and DCQ use. | Provide educational session |

## **8. DCQ Use and Workflow Integration**

| Item | Action | Operationalisation |
| --- | --- | --- |
| Who administers the DCQ | Define who applies the tool (e.g., nurses, champions). | Team 1: All nurses and nurse assistants Team 2, 3, 5: Contact local champions, local champions will use the DCQ. Team 4, 6: Use the DCQ together with local champions. |
| When to screen | Determine screening triggers (e.g., behavioural changes, gut feeling). | Behavioural changes, gut feeling, clinical judgment |
| Documentation | Decide how and where to record DCQ scores (paper, EHR). | All teams: 1. Report symptoms in EHR.  2. Administer DCQ on paper |
| Materials | Ensure DCQ forms are easily accessible (e.g., via shared folders, physical location). | All teams had DCQ forms in the office and pocket cards with them. |

## **9. Monitoring and Feedback**

| Item | Action | Operationalisation |
| --- | --- | --- |
| Plan evaluations | Schedule team check-ins or evaluations after initial implementation. | All teams had one meeting approximately one month post educational session to discuss initial reactions and questions. |
| Track progress | Encourage note-taking or logbook use for capturing key lessons and questions. | Discussed during champion meetings. |

## **10. Communication and Support**

| Item | Action | Operationalisation |
| --- | --- | --- |
| Contact person | Assign a central contact (e.g., researcher or coordinator) for implementation questions. | KO was central contact. |
| Channels | Decide how teams can reach support (e.g., email, messaging, phone). | Contact info was given to all teams. |

## **11. Research and Consent Procedures**

| Item | Action | Operationalisation |
| --- | --- | --- |
| Client selection | Define how clients are identified and approached for consent. | Champions identified clients with (perceived) cognitive decline and approached them for consent during a home visit. |
| Informed consent | Determine who distributes and collects signed consent forms. | All teams: champions. |
| Data feedback | Decide how completed DCQs and outcomes are reported to the research team. | All teams: completed DCQ forms collected at the office and given to researcher during final meeting. |

This template the team-specific action plans used in the study and is intended to provide transparency regarding the design and execution of the intervention. It also serves as a model for other organizations seeking to implement the DCQ or similar tools in community care settings.

**Additional file 4. Educational session**

*Manuscript:* District nurses’ experiences with the implementation of an evidence-informed triage instrument for delirium in home care; a qualitative study

This educational session was used to implement the Delirium Care Questionnaire (DCQ) in district nursing teams. It was developed by implementation experts of the University Elderly Care Network of the University Medical Centre Groningen (UECN-UMCG).

**Initial Situation**
General knowledge about the prevention, recognition, and treatment of delirium was insufficient. Nurses and nurse assistants were unfamiliar with the Delirium Care Questionnaire (DCQ) as a screening tool.

**Objective**
To enable district nurses and nurse assistants to consciously and competently use the DCQ to support early identification of delirium in older adults with dementia living at home.

**Training Structure**

| Learning Objective | Learning Activities | Methodology | Materials | Duration |
| --- | --- | --- | --- | --- |
| Refresh basic knowledge of delirium (prevention, recognition, treatment) | Independent self-study (preferably in pairs), using their organization’s LMS or UNO platform | E-learning | LMS/UNO, www.watdoejebijdelier.nl | ~4 hours (pre-training) |
| Introduce session and create psychological safety | Welcome, ground rules, agenda | Plenary discussion | None | 10 min |
| Reinforce prior knowledge | Group game “What am I?” using delirium-related terms | Group game and short debrief | Post-its | 10 min |
| Theoretical introduction to DCQ | Presentation and discussion on background, purpose, and structure of the DCQ | Interactive lecture | PowerPoint, DCQ handout | 20 min |
| Practice applying the DCQ | Watch case video, discuss as a group | Video-based case discussion | USB video, projector | 10 min |
| Simulate use of the DCQ in practice | Role-play in trios: observer, informal caregiver, nurse | Simulation | DCQ form, notepad | 20 min + debrief |
| Peer feedback and self-reflection | Peer assessment of DCQ use | Trio discussion | Self-rating scale | 10 min |
| Discuss implementation challenges and opportunities | Reflective discussion on potential barriers and facilitators | Plenary dialogue using visual metaphor (cards) | Metaphor cards | 10 min |
| Team-based planning | Develop action points for integrating DCQ in routine care | Small-group discussion | Flipchart or worksheet | 15 min |
| Evaluation | Share key takeaways and reflections | “Clothesline and clothespins” exercise | String and pins | 1 hour (third session) |

**Evaluation Goal**
- At least 60% of participating professionals should use the DCQ in clinical practice.

**Additional file 5. Interview guide**

*Manuscript:* District nurses’ experiences with the implementation of an evidence-informed triage instrument for delirium in home care; a qualitative study

This interview guide was used to collect data on the experiences of the participating teams with the DCQ through semi-structured interviews with the nurses. We developed this semi-structured interview guide based on the CFIR and the RE-AIM. The original interview guide was made in Dutch. We have adapted it to English for informative purposes.

**Introduction**
We would like to talk with you about the Delirium Care Questionnaire (DCQ) and the process of working with it. Our aim is to understand what works well with the DCQ, what doesn’t, and how the implementation process went. You can share anything you feel is relevant. The goal of this study is to explore whether the instrument adds value and is usable in daily practice. You represent daily practice, and the instrument should align with your reality. This is not a performance evaluation. It doesn’t matter if you rarely used the tool or if you weren’t motivated to use it. That would indicate a limitation in the tool, implementation method, or context.

**Start: Open Questions**
- Please think of two positive and two negative thoughts that come to mind regarding the DCQ.
- Can you elaborate?
Ask follow-up questions based on the input of the participants.

Ask the following additional questions if the topics have not been covered yet:

**Preparation**

- How did you experience the implementation of the DCQ?

- Have you been involved in previous projects where new practices were introduced in your organization?

- Do you feel that the implementation approach for the DCQ was different? Why or why not?

- What differences did you notice, or why do you perceive no difference?

- Did you have enough freedom to adapt the use of the DCQ to your team's needs?

- Did you use the provided materials (e.g., pocket card, office binder)? If yes, how?

- Do you have suggestions for making it easier to read and use the materials?

**Experiences and Outcomes**

- Was the DCQ used after the interim evaluation?

- How often did you personally use the DCQ?

- How often was the DCQ used by your team?

- How did this go?

- Why did you use the DCQ at that time?

- How often did the DCQ yield a positive score?

- What was it like working with the DCQ?

- How was the DCQ received by your team?

- Would you like to continue using the DCQ after the pilot? Why or why not?

- What improvements could be made to the DCQ?

- What aspects of the DCQ should remain unchanged?

- Do you think you always recognize delirium?

- - If yes/no, has this changed since the project?

- - What contributed to this change? (DCQ, training, materials?)

**Execution**

- How feasible is it to use the DCQ in district nursing practice?

- With whom did you administer the DCQ (e.g., informal caregiver, key nurse)? Why?

- Did anything unexpected happen when using the DCQ? Please elaborate.

- Which items were difficult to complete for you or the informal caregiver?

- What follow-up steps did you take based on the DCQ result?

- Did anything unexpected happen during the use of the DCQ? Can you explain?

**Suitability for Community Use**

- How suitable is the DCQ for use with older adults in the community?

- - I can use it with clients with diverse care needs.

- How suitable is the DCQ for use by home care nurses?

- - It is inexpensive.

- - It ultimately saves me time.

- - I can use it in daily care.

- What influence does the DCQ have on client outcomes?

**Additional Influencing Factors**

- How was the DCQ discussed within the team?

- How did the DCQ influence communication with colleagues or other disciplines such as GPs?

- How did your organization influence the use of the DCQ?

- Should anything change in the organization to support DCQ use? If yes, what?

- Did the way your team works affect DCQ use? If yes, how?

- Were there clients for whom you wanted to use the DCQ but didn’t? Why?

**Future Outlook**

- In an ideal situation, how would you approach delirium recognition in the future?

- What would be needed to achieve this?

**Closing the interview**

Thank you very much for sharing your experiences and thoughts today. Before we finish, is

there anything else you would like to add or discuss that we haven’t touched on yet?

To explain the next steps: within a week, you will receive a short summary of today’s interview.

Later on, we will transcribe the full interview and use it — along with other interviews and data —

to inform our analysis and write up the study results.

Do you have any questions for us about the study or the next steps?

**Additional file 6. Team-specific details**

*Manuscript:* District nurses’ experiences with the implementation of an evidence-informed triage instrument for delirium in home care; a qualitative study

|  | Team A | Team B | Team C | Team D | Team E | Team F |  |
| --- | --- | --- | --- | --- | --- | --- | --- |
| **Team characteristiscs** | | | | | | | |
| Team size nurses | 10 | 5 | 12 | 12 | 12 | 20 |  |
| Number of champions | 2 | 2 | 4/1 | 4 | 3/1 | 3 |  |
| Team size clients | 36 | 20 | 40 | 50 | 60 | 50 |  |
| Participating clients | 5 | 4 | 6 | 3 | 0 | 10 |  |
| Times DCQ used | - | 1 | - | 1 | - | 1 |  |
| **Nurses participating in interviews characteristics** | | | | | | |  |
| Total | 2 | 2 | 2 | 4 | 2 | 3 |  |
| Female | 50% | 100% | 100% | 100% | 100% | 100% |  |
| Community nurse | 2 | 1 | 1 | 1 | - | 1 |  |
| Certified nurse aid | - | 1 | 1 | 3 | 1 | 2 |  |
| Team manager | - | - | - | - | 1 | - |  |
